# Hysteroscopic Findings in Patients with A History of Two Implantation Failures Following *In Vitro* Fertilization

**Published:** 2012-06-19

**Authors:** Ashraf Moini, Kiandokht Kiani, Firouzeh Ghaffari, Fatemeh Hosseini

**Affiliations:** 1 Department of Endocrinology and Female Infertility, Reproductive Biomedicine Research Center, Royan Institute for Reproductive Biomedicine, ACECR, Tehran, Iran; 2 Department of Obstetrics and Gynecology, Faculty of Medicine, Tehran University of Medical Sciences, Tehran, Iran

**Keywords:** Hysteroscopy, Uterine Pathology, *In vitro* Fertilization

## Abstract

**Background:**

This study was designed to evaluate the incidence of uterine pathologies in infertile women with a history of two implantation failures after *in vitro* fertilization (IVF) and estimate the effect of hysteroscopic correction on achieving a pregnancy in these patients.

**Materials and Methods:**

The retrospective study population included 238 infertile women attended the outpatient infertility clinic between November 2007 and December 2008. Patients with at least two previous IVF failures were eligible for this study. All patients had normal findings on hysterosalpingography performed prior their first attempt for IVF. Standard transvaginal ultrasonography and diagnostic hysteroscopy were performed in patients before the subsequent IVF attempt.

**Results:**

Out of 238 patients with previous IVF failure who underwent hysteroscopic evaluation, 158 patients (66.4%) showed normal uterine cavity. Abnormal cavity was found in 80 patients (33.6%). We found polyp as the most common abnormality (19.7%) in the patients with previous history of IVF failure. The pregnancy rate was similar between IVF failure patients who treated by hysteroscopy for a detected uterine abnormality (24.6%) and similar patients with normal uterine cavity (21.2%) in hysteroscopic examinations.

**Conclusion:**

The intrauterine lesions diagnosed by hysteroscopy in patients with previous IVF failure ranges from 0.8%-19.7%. Correction of abnormalities such as myoma and polyp showed good outcome, similar to that achieved in patients with a normal hysteroscopy.

## Introduction

The success of *in vitro* fertilization (IVF) treatment depends on embryo quality, uterine receptivity and uterine integrity. Benign endometrial pathologies, such as endometrial adhesions, polyps, hyperplasia, endometritis and uterine mullrian abnormalities have an adverse effect on endometrial receptivity and consequently in pregnancy rate (1-4).

Uterine abnormalities can be diagnosed by vaginal sonography, hysterosalpingography, sonohysterography and hysteroscopy (5, 6). The essential role of hysteroscopy in the diagnosis of intra uterine pathologies is emphasized, especially in infertile patients (7). However, hysteroscopy does not utilize as a routine investigation for infertile women and there are different guidelines about performing the hysteroscopy. Some researchers believe that hysteroscopy is necessary for treatment of suspicious uterine pathologies (8), whereas the others have found no benefit in fertility enhancement after treatment of uterine anomalies (9).

## Materials and Methods

The study population included 238 infertile women attended Royan reproductive research centre, Iran, between November 2007 and December 2008. Patients with at least two previous IVF-embryo transfer (ET) failures were eligible for participation in this study. Approval from the institutions ethics committee had been obtained before the study and all patients signed informed consent to include in the research. All participants were guaranteed confidentiality and anonymously.

All patients had normal findings on hysterosalpingography performed prior their first attempt for IVF. Standard transvaginal ultrasonography and diagnostic hysteroscopy were performed in patients before the subsequent IVF attempt. The results of hysteroscopy and treatment outcome were obtained by reviewing the patients’ records.

All hysteroscopies and endometrial procedures were performed during the follicular phase of the cycle. The diagnostic hysteroscopies were performed under general anesthesia using a hysteroscope (Karl Storz company, Germany), which it had a 30˚ view with 2.9 mm Bettochi continuous flow sheath. The uterine distention was performed with glycine using an electronic pomp (Hysteromat; Karl Storz). Surgical video assisted hysteroscopy was performed by using a mono polar electric resectoscope (Karl Stroz) with an outer diameter of 9 mm diameter.

In patients with uterine pathology, suitable treatment was performed at the same time. If polyp, sub mucous myomas or adhesions were found, immediate hysteroscopic resection or adhesion lysis were performed. Septae were excised by scissors or resectoscope. In patients with polyps at size of more than 2 cm or sub mucous myoma, cervical dilation was performed.

After examination of the cervical canal and uterine cavity, endometrial tissue samples were obtained if necessary (only for patients with sub mucosal myoma or endometrial polyp). All the samples were fixed in formaldehyde and sent for pathology. The findings were classified as normal if the endometrium was regular, or there were no pathologic findings.

Stimulation protocol in all patients was according to the standard long protocol. All patients received oral contraceptive pills (OCP) from the second or third day of menstrual cycle which it continued by Busereline (500 μg, Suprefact; Aventis Pharma Deutshlan, Frankfurt, Germany), via subcutaneous injection starting on the 21st day of their menstrual cycles. Down regulation was confirmed by linear endometrium in ultrasonography. Gonadotrophin stimulation was started fourteen days after subcutaneous GnRH agonist injection with recombinant FSH (Gonal F, Serono, Switzerland) 150 IU, daily. The dose and duration of FSH treatment were adjusted by monitoring follicular development through an ultrasound and estradiol levels. The goal of ovarian stimulation was to achieve an average of two ovarian follicles with a mean diameter of ≥17 mm on the day of human chorionic gonadotropin (hCG) administration. HCG (Choriomon; IBSA, Lugano, Switzerland) 10000 IU was intramuscularly injected and oocyte retrieval was performed 34-36 hours later.

Maximum of four embryos were transferred 2 to 3 days after oocyte retrieval. The vaginal progesterone (Aburaihan Co., Tehran, Iran) 400 mg twice a day was used as luteal-phase support and continued until the tenth weeks of gestation.

All statistical were performed by means of the SPSS program (version 13). Statistical comparison between groups was performed by chi-square test when appropriate. A p value of < 0.05 was considered significant. We used number and percentage for expression of categorical or descriptive data.

## Results

The study population included 238 infertile women with at least two previous IVF failures. The age of patients ranged between 19 and 47 years (mean 34.5 ± 5.6 years) and duration of infertility ranged from 1 to 27 years (mean 11.03 ± 5.7 years). Ninety seven percent of patients had primary and 3% secondary infertility.

Out of 238 patients with previous IVF failure that underwent hysteroscopic evaluation, 158 patients (66.4%) showed normal uterine cavity. Abnormal cavity was found in 80 patients (33.6%). Our results showed uterine polyp as a most common abnormality in patients with previous history of IVF failure (19.7%). Structural abnormalities (such as arcuate, unicorn and septate uterus) (10.9%) and Sub mucosal myoma (2.1%) were the other important findings in these patients, respectively (Table 1). Adhesion bands (Asherman syndrome) were observed in 0.8% of patients. Tissue samples were only obtained in patients with sub mucosal myoma or uterine polyp. All theses abnormalities were treated during hysteroscopy without any difficulty.

Table 2 summarizes the outcomes of the subsequent IVF cycles in women with a detected and treated uterine abnormality. Clinical pregnancy rate was also measured based on each specific abnormality. We found higher pregnancy rate in women with treated uterine sub mucous myoma compared the other types of uterine abnormalities. However, this rate did not reach to statistically significant level.

**Table 1:** Clinical findings reported by hysteroscopy and histology in patients with previous IVF failure


Finding	Cases suspected by hysteroscopy	Cases confirmed by histopathology	Cases did not confirm by histopathology	Cases who had no pathology specimen

**Normal**	158 (66.4 %)	35 (13.5%)	2 (0.8%)	121 (51%)
**Endometrial polyp**	47(19.7 %)	34 (14.3%)	5 (2.1%)	8 (3.4%)
**Structural abnormality**	26(10.9%)	0	0	26 (11%)
**Sub mucosal myoma**	5 (2.1 %)	4 (1.7%)	0	1 (0.4%)
**Asherman syndrome**	2 (0.8 %)	0	0	2 (0.8%)


**Table 2:** Outcomes of the subsequent IVF cycles in women with a detected and treated uterine abnormality


Type of uterine abnormality	No. of cases (%)	No. of cases who missedduring follow up	Clinical pregnancy rate

**Endometrial polyp**	47 (19.7%)	14	8/33 (24.2%)
**Structural abnormality (Arcuate, Unicorn, Septum)**	26 (10.9%)	8	4/18 (22.2%)
**Sub mucosal myoma**	5 (2.1%)	1	1/4 (25%)


The pregnancy rate was also compared between repeated IVF failure patients who treated by hysteroscopy for a detected uterine abnormality and similar patients with normal uterine cavity in hysteroscopic examinations. Our results revealed no significant pregnancy rate between two groups (Table 3).

**Table 3:** Comparison between patients with previous IVF failure who treated by hysteroscopy and similar patients with normal uterine cavity in hysteroscopic examinations


	Non pregnant	Pregnant	Total

**Normal**	82 (78.8%)	22 (21.2%)	104
**Abnormal**	43 (75.4%)	14 (24.6%)	57
**P value**	0.620		


## Discussion

The role of hysteroscopy for assessing the uterine integrity in patients with good quality embryos who fail to conceive has been known for many years (14, 15). Even when no abnormality is found with the other diagnostic tools, a significant percentage of patients have been found to carry subtle intrauterine pathologies that may diminish the success of IVF treatments (16, 17).

The aim of present study was to report the uterine pathologies in patients with previous IVF failure which diagnosed only by hysteroscopy. We did not have access to all pathology results because some results were not reported to this center by other laboratories or patients. Therefore, the results of hysteroscopy were reported for measuring the objective of the study. The results of pathology were presented in table 1, only as descriptive findings.

At present study, in 80 patients (33.6%) of the population study, an abnormality on hysteroscopy was identified. The prevalence of polyps and submucous leiomyomata in patients with previous IVF failures were about 19% and 2%, respectively. Theses lesions are the most common structural pathologies in the uterine cavity; however, their prevalence in patients with repeated failure of IVF-ET is not known, clearly. Oliveira et al. has reported the prevalence about 4% and 18% for polyps and submucous leiomyomata, respectively, in patients with repeated failure of IVF-ET (6). Uterine polyp was also as a most common abnormality in patients with previous history of IVF failure (19.7%). Polyp may cause infertility in 5 -10% and recurrent abortion in 15- 50% of all cases (18, 19). It seems that these intrauterine lesions either have been missed during previous investigations, or newly developed because of treatment cycles or embryo transfer (11).

We found an improved clinical pregnancy rate (more than %20) in new IVF cycle of women with abnormal hysteroscopic findings. Although, there were no significant difference about the pregnancy rates between women with abnormal hysteroscopic findings and women with normal uterine cavity; this pregnancy rate after two previous IVF failures will be acceptable and satisfying. Demirol and Gurgan also could not find any significant difference between patients who had normal or abnormal hysteroscopic findings (11).

At present study, we did not compare the pregnancy rate between IVF failure patients who had hysteroscopy and whom with no hysteroscopy. However, in one study, there was significant difference in the clinical pregnancy rates between these two groups (11).

## Conclusion

Diagnostic hysteroscopy is a valuable tool in detecting intra uterine pathologies, especially polyp and myoma. The frequency of intrauterine lesions diagnosed by hysteroscopy in patients with previous IVF failure ranges from 0.8%-19.7%. Correction of abnormalities such as myoma and polyp showed good outcome, similar to that achieved in patients with a normal hysteroscopy. Further researches with adequate sample size are recommended for approving these results.
